# Investigating Embryonic Expression Patterns and Evolution of *AHI1* and *CEP290* Genes, Implicated in Joubert Syndrome

**DOI:** 10.1371/journal.pone.0044975

**Published:** 2012-09-24

**Authors:** Yu-Zhu Cheng, Lorraine Eley, Ann-Marie Hynes, Lynne M. Overman, Roslyn J. Simms, Amy Barker, Helen R. Dawe, Susan Lindsay, John A. Sayer

**Affiliations:** 1 Institute of Genetic Medicine, International Centre for Life, Newcastle University, Central Parkway, Newcastle upon Tyne, United Kingdom; 2 Biosciences: College of Life and Environmental Sciences, University of Exeter, Stocker Road, Exeter, United Kingdom; University of Tampere, Finland

## Abstract

Joubert syndrome and related diseases (JSRD) are developmental cerebello-oculo-renal syndromes with phenotypes including cerebellar hypoplasia, retinal dystrophy and nephronophthisis (a cystic kidney disease). We have utilised the MRC-Wellcome Trust Human Developmental Biology Resource (HDBR), to perform in-situ hybridisation studies on embryonic tissues, revealing an early onset neuronal, retinal and renal expression pattern for *AHI1.* An almost identical pattern of expression is seen with *CEP290* in human embryonic and fetal tissue. A novel finding is that both *AHI1* and *CEP290* demonstrate strong expression within the developing choroid plexus, a ciliated structure important for central nervous system development. To test if *AHI1* and *CEP290* may have co-evolved, we carried out a genomic survey of a large group of organisms across eukaryotic evolution. We found that, in animals, *ahi1* and *cep290* are almost always found together; however in other organisms either one may be found independent of the other. Finally, we tested in murine epithelial cells if Ahi1 was required for recruitment of Cep290 to the centrosome. We found no obvious differences in Cep290 localisation in the presence or absence of Ahi1, suggesting that, while Ahi1 and Cep290 may function together in the whole organism, they are not interdependent for localisation within a single cell. Taken together these data support a role for *AHI1* and *CEP290* in multiple organs throughout development and we suggest that this accounts for the wide phenotypic spectrum of *AHI1* and *CEP290* mutations in man.

## Introduction

Joubert syndrome and related diseases (JSRD) are a group of inherited ciliopathies, characterised by a cerebello-retinal-renal phenotype. The brain phenotype is a developmental midbrain malformation leading to cerebellar vermis hypoplasia or aplasia, and seen in brain MRI imaging as a “molar tooth sign” [1]. Other structural brain defects including hypoplasia of the corpus callosum and occipital meningoencephalocele have been reported [2], [3].

Retinal dysplasia and degeneration occur in a proportion of patients and may lead to progressive blindness [1], [4], [5]. Ocular coloboma may also be a feature [6]. Renal disease is variable, with nephronophthisis [7] and multicystic dysplasia [8] as reported phenotypes, which may lead to end stage renal failure. Consistent with JSRD as a ciliopathy are the findings of polydactaly and liver fibrosis [9], [10].

In keeping with the clinical heterogeneity of JSRD, 16 causal genes, have been identified in patients. These include *INPP5E* [11], *TMEM216*9 [12], *AHI1* [7], [13], [14], *NPHP1* [15], *NPHP6* (*CEP290*) [3], *TMEM67* [16], *RPGRIP1L* [8], *ARL13B* [17], *CC2D2A* [18], *CXORF5* [19], *TTC21B* [20], *KIF7* [21], *TCTN1* [22], *TMEM237* [23], *CEP41* [24] and *TMEM138* [25]. Defects in these genes produce phenotypes that may be termed ciliopathies, given that the protein products encoded by all of these genes have been localised in the basal body, centrosome or primary cilium [10], a highly conserved cellular organelle, central to the regulation of cellular signalling pathways [26].

Mutations in *AHI1* and *CEP290* are both a frequent causes of Joubert syndrome and genetic variants in both genes may act as modifier alleles, especially in regard to a retinal and CNS phenotype [27], [28]. Mutations in *AHI1* (Abelson-helper integration site-1) are the most common genetic cause of JSRD, accounting for 12% of cases and 20% of individuals with Joubert syndrome and Leber's congenital amaurosis [13], [14], [29], [30], [31].

*AHI1* is highly conserved throughout evolution and encodes the Ahi1 protein (also known as Jouberin). We have previously demonstrated that Ahi1 localises to centrosomes/basal bodies of renal epithelial cells, and that it interacts with the nephrocystin-1 protein [32]. At a genomic level, there is evidence for *AHI1* mutations/polymorphisms having an oligogenic effect and modulating the phenotype. For example Tory *et al*. describe 5 patients with homozygous *NPHP1* mutations together with a R830W mutation in *AHI1*, which leads to a more severe central nervous system defect [27]. Similarly, the relative risk (RR) of a retinal defect associated with nephronophthisis was significantly increased (RR = 7.5 (95% CI 4.0–11.2)) in the presence of the R830W *AHI1* mutation [31].

Mutations in *CEP290* may cause a wide spectrum of human disease ranging from isolated Leber's congenital amaurosis to JSRD, Meckel syndrome (MKS) and Bardet-Biedl syndrome (BBS) [33], [34], [35]. Renal disease is common in patients with *CEP290* mutations, and may be secondary to NPHP and renal cortical cysts [3], [36].

*CEP290* mutations account for around 50% of cases of JSRD with renal and retinal disease [3], [27], [36], [37], [38], [39], [40]. Some additional phenotypes in patients with *CEP290* mutations have included occipital encephaloceles [3] and septal heart defects [37]. *CEP290* mutations are a common cause of isolated Leber's congenital amaurosis, accounting for ∼20% of patients [39], [41].

*CEP290* is highly conserved throughout evolution and encodes the Cep290 protein (also known as nephrocystin-6) [3]. We have previously demonstrated that Cep290 localises to centrosomes/basal bodies of renal epithelial cells and that it interacts with ATF4 [3]. Additional *CEP290*/Cep290 interactions include a genetic interaction with *CC2D2A* [18] and protein complex interactions with CP110 and Rab8a [42]. In a similar manner to patients with *NPHP1* mutations, there is some evidence that alleles of *AHI1* may also modify the neurological phenotype of patients with *CEP290* mutations [28].

Given this evidence implicating an important role for *CEP290* and *AHI1* in modulating brain, eye and other phenotypes in JSRD, we sought to identify the pattern of both *AHI1* and *CEP290* expression in kidney and central nervous system during development. Using human embryonic and fetal tissue we describe the expression pattern of *AHI1* and *CEP290* during stages of human renal and brain development (Table 1).

**Table 1:** Overview of renal and brain development time points in human embryogenesis.

Carnegie Stage	Approximate age (dpc)	Kidney development	Brain development
CS12	26	The mesonephros begins to form	Formation of neural tube with primordial brain at rostral end consisting of three primary brain vesicles (primary forebrain, midbrain and hindbrain)
CS14	32	The metanephros starts to form deep in the pelvic region; ureteric buds are identifiable; glomeruli are visible in the mesonephros	Forebrain is divided into telencephalon and diencephalon, optic cup usually present, rhombomeres 1–8 visible in hind brain
CS15	33		Cerebral hemispheres start to develop in telencephalon
CS16	37	Metanephros ascends through the abdominal cavity	
CS17	41	Glomeruli now visible in metanephros	First appearance of choroid plexus in telencephalon
CS19	47.5		Early stages of cerebellum formation
CS20	50.5	Collecting ducts now visible in metanephros	
CS21	52		Cortical plate appears in cerebral hemispheres
CS22	54	Mesonephros starts to degenerate	
CS23	56		Cortical plate present throughout cerebral cortex; external germinal layer visible in the cerebellum
9 PCW	57–63	Distinct regions become visible in metanephros	

To determine whether *AHI1* and *CEP290* may have co-evolved, we performed a genomic survey of a large group of organisms across eukaryotic evolution. We also explored whether Ahi1 was required for recruitment of Cep290 to the centrosome.

Our novel data emphasises the important and extensive role of *AHI1* and *CEP290* in vertebrate development, from essential cellular signalling organelles, such as the primary cilium, to complex organ systems such as the brain and kidney.

## Materials and Methods

### Ethics statement

This study was conducted with full ethical approval. For human embryonic and fetal tissue samples, the samples were collected with appropriate maternal consents and ethical approval by the Newcastle and North Tyneside 1 Research Ethics Committee, UK.

### Human tissue in-situ hybridisation

Human embryonic and fetal tissues were obtained from the MRC/Wellcome Trust-funded Human Developmental Biology Resource (http://www.hdbr.org; [43]). In situ hybridisation was performed on human embryo paraffin sections as previously described [44] with some modifications. Briefly, sections were dewaxed in xylene, gradually hydrated in decreasing ethanol concentrations before incubation in Proteinase K (20 µg/ml) at room temperature (RT), followed by fixation in 4% paraformaldehyde in PBS. Background was reduced by treating with 0.1 M Triethanolamine pH 8. Sections were air dried and probe added (300 ng labeled probe per 100 ul of Dig Easy Hyb mix (Roche)) at 68°C overnight. The next day sections were washed once in 5× SSC then once in 2× SSC at 60°C then incubated with anti-digoxigenin AP Fab fragments (Roche) diluted 1∶1000 at 4°C overnight. Sections were then washed and expression detected using NBT/BCIP (20 ul/ml Roche) in 0.1 M Tris/0.1 M NaCl (pH 9.5) in the dark at room temperature. Developing was stopped by rinsing slides first in 0.1 M Tris/0.1 M NaCl (pH 9.5) then in deionised H_2_O. Sections were mounted using Aquamount. Samples from Carnegie stage (CS) 12 (∼26 days post conception (dpc)) to 9 weeks post conception (PCW) were used (CS12 (N548); CS14 (N663, N7120); CS15 (N734); CS16 (N218); CS17 (N1247); CS19 (N470); CS20 (N590); CS22 (N225, N2188); CS23 (N300); 9 PCW (N477, N9223)). Sections from 13 different embryos/fetuses were used in total.

*In situ* hybridisation probes for human expression studies of *AHI1* were generated using PCR and subcloning into pGEMTeasy vectors. All clones were sequenced to confirm orientation with respect to T7 and SP6 promoters. Both sense and antisense RNA probes were produced by *in vitro* transcription from the T7 and SP6 RNA polymerase sites respectively of the pGEM-T Easy vector. Probes were generated from two regions of the *AHI1* gene (BC094800), nucleotides 1 to 276 (in 5′UTR) and nucleotides 2105 to 2635 (in 3′UTR) using the “Riboprobe” system (Promega). Both probe sets gave similar patterns however the 5′UTR probe set gave a higher background in preliminary experiments (data not shown). The 3′UTR probe set was used for all the experiments reported here.

*In situ* hybridisation probes for human expression studies of *CEP290* were generated from two regions of *CEP290* (NM_025114) using the “Riboprobe” system (Promega) as above: nucleotides 675 to 1381 and 6265 to 6924 (both in the coding sequence). Although a similar pattern was detected with both antisense probes, there was a higher background with the 675 to 1381 nucleotide sense probe (data not shown). The 6265 to 6924 nucleotide probe set was used for all the experiments reported here.

Antisense probes for sonic hedgehog (*SHH*, NM_000193) were used as a positive control. The *SHH* probe, which contains nucleotides from 460 to 950, showed the expected specific staining in the spinal cord and notochord (data not shown). Antisense RNA probe of developing brain homeobox 1 (*DBX1*, NM_001029865) transcribed from nucleotides 308 to 830 which shows specific staining at the sulcus limitans of spinal cord was also used as an experimental control (data not shown). Expression patterns were analysed using the Axioplan 2 imaging system (Zeiss).

### Sequence analysis

Putative Ahi1 and Cep290 orthologues were identified using a combination of reciprocal best BLASTP and PSI-BLAST, with human Ahi1 (isoform a, NP_001128302.1) and Cep290 (NP_079390.3) as the query sequences [45], [46]. The initial Ahi1 search generated a very large number of false positives, which were only similar in terms of WD40 repeats. To overcome this problem, we performed further searches using human Ahi1 amino acids 1–545. These protein sequences were used to query the non-redundant predicted proteomes of 44 organisms (33 flagellate, 11 non-flagellate) chosen to represent a wide evolutionary spread of eukaryotes. Searches were carried out using the NCBI stand-alone BLAST+ application (version 2.2.25+, [47]) against BLAST-formatted genomes from a variety of sources (see Supplementary Figure S1). We used the following criteria to define a putative Ahi1 or Cep290 orthologue: 1) An e-value less than 1e-05 (BLASTp) or 1e-10 (PSI-BLAST), 2) A reciprocal BLAST which returns the original sequence, and 3) Manual examination of sequences and alignments constructed using MAFFT [48].

### Cell culture, siRNA, and immunofluorescence using mIMCD-3 cells

Mouse inner medullary collecting duct (IMCD-3) cells were cultured in DMEM/Ham's F12 supplemented with 10% fetal calf serum (Sigma–Aldrich Co. Ltd., Poole, UK). For functional analyses, passage 16–17 IMCD3 cells were cultured on 13 mm glass coverslips. Cells were transfected with a pool containing 100 pmol of each of four siRNA duplexes (OnTargetPlus SMARTpool, Dharmacon) against mouse Ahi1 at 60–70% confluency using Lipofectamine 2000 according to our previously established methods [49]. The medium GC non-targeting control (Invitrogen) was used as a negative control. siRNAs were as follows: oligo 1 5′-GGUCAAAAGACGAUCGCUA-3′, oligo 2 5′-UGAAGUUAGCCGCCGUGUAA-3′, oligo 3 5′-GCUAAAUGUCGUCGAGGUU-3′, oligo 4 5′-GUGAAACACUGUAUCGAGA-3′. As a further control, siRNA studies were repeated with two individual siRNA oligonucleotides (numbers 2 and 3, above, data not shown). We have previously characterised these duplexes in this same cell line and demonstrated successful siRNA knockdown [49]. Transfected cells were identified using co-transfection with siGLO red (Dharmacon). Transfection efficiency was estimated at 75–80%. Immunofluorescence was carried out 72 hours after transfection using anti-Cep290 (Abcam, ab84870) and anti-gamma tubulin (clone GTU-88, Sigma Aldrich) followed by goat anti-mouse AlexaFluor 488 and 647 (Molecular Probes). Samples were visualised using a Zeiss LSM510 confocal microscope controlled by LSM examiner software (Carl Zeiss). Images were processed in ImageJ to produce maximum intensity projections.

## Results

### *AHI1* expression during human brain and kidney development

There has been no previous documentation of *AHI1* spatial expression during development in human tissue. Previous characterisation has been limited to hybridisation to RNA northern blot showing expression in fetal brain, kidney, liver and lung [13], [14] and Western blotting, demonstrating Ahi1 protein expression in fetal cerebellum and spinal cord [50].

**Figure 1:**
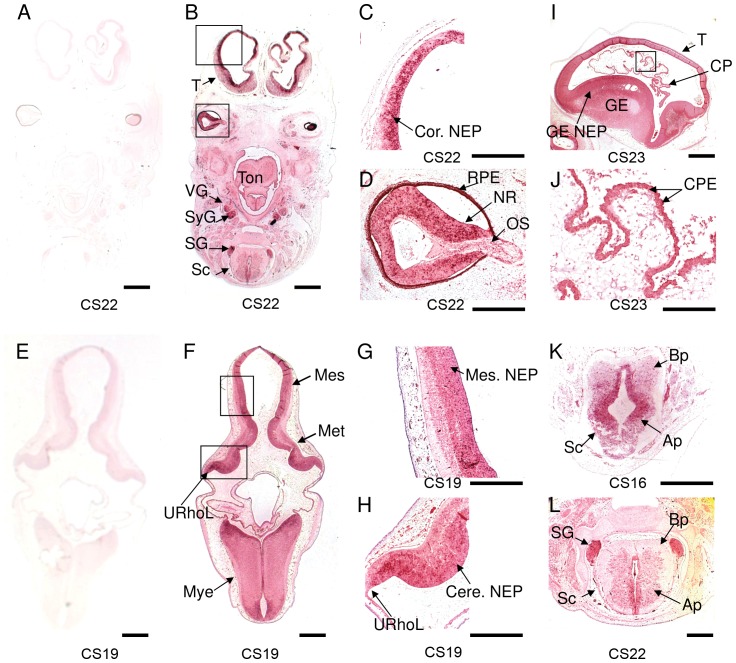
*AHI1* is expressed in the developing cerebellum, spinal cord, choroid plexus and eye. (A, E) Negative control hybridisation with *AHI1* sense RNA probe and (B–D) hybridisation with *AHI1* antisense RNA probe to transverse sections at CS22. *AHI1* transcripts are abundant in the developing telencephalon, especially the neuroepithelium (C) and neural retina (D). Prominent expression is seen in (F–H) metencephalon (including cerebellum), myelencephalon and mesencephalon at CS19. Strong expression is detected in the neuroepithelium of the developing mesencephalon and cerebellum (G, H). Within the developing ventricles, strong expression is seen in the choroid plexus epithelium (I, J) and the neuroepithelium of the ganglionic eminences at CS23 (I). Prominent staining is demonstrated within (K) the alar plate of spinal cord at CS16 and (L) CS22 and in the spinal ganglia. Ap, alar plate; Bp, basal plate; GE, ganglionic eminence; NEP, neuroepithelium; Cor.NEP: cortical neuroepithelium; Mes.NEP: mesencephalic NEP; Cere.NEP: cerebellar NEP; CP: choroid plexus; CPE, choroid plexus epithelium; Mes, mesencephalon (midbrain); Met, metencephalon; Mye, myelencephalon; NR, neural retina; OS, optic stalk; URhoL, upper rhombic lip; RPE, retinal pigment epithelium; Sc, spinal cord; SG, spinal ganglion; SyG, sympathetic ganglion; T, telencephalon; Ton, tongue; VG, vagal ganglion. Scale bars: A, B, E, F = 2 mm; C, D, G, H, K = 500 µm; I = 1 mm; J = 250 µm; L = 500 µm.

Using specific probes targeted towards nucleotides 2105 to 2635 of *AHI1* mRNA we observed a strong and specific pattern of expression in the brain and spinal cord from CS16 to CS23 (Figure 1; see also Table 1 for developmental overview). *AHI1* expression was abundant in the developing telencephalon (Figure 1B), especially the neuroepithelium, and in the neural retina (Figure 1C, D respectively). Strikingly, we observed expression within the choroid plexus (Figure 1I, J). Prominent expression was also seen in the mesencephalon and throughout the developing hindbrain (metencephalon and myelencephalon, Figure 1F–H) including the rhombic lip of the developing cerebellum (Figure 1H). There was particularly prominent staining within the alar plate of the spinal cord as well as in the spinal and other ganglia of the peripheral nervous system (Figure 1K, L). *SHH* and *DBX1* antisense probes were used as positive controls and the expected patterns of expression were seen in the developing spinal cord (data not shown).

**Figure 2:**
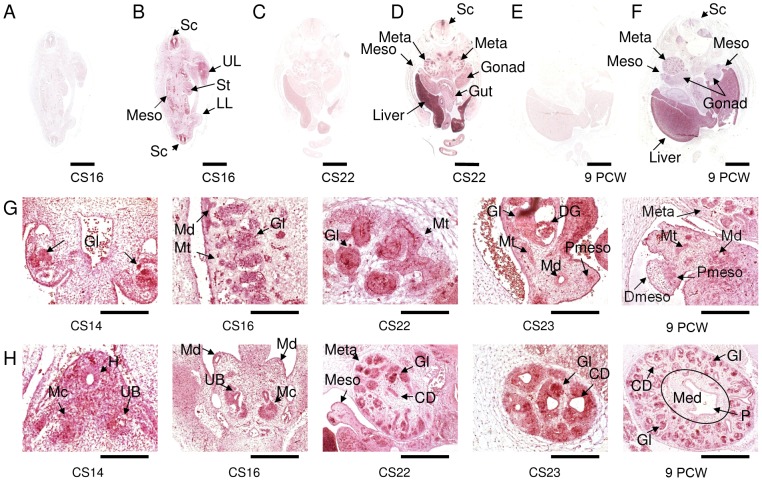
*AHI1* expression during nephrogenesis. (A, C, E) *AHI1* sense RNA probe and (B, D, F) *AHI1* antisense RNA probe hybridised to sections from (A, B) CS16; (C, D) CS22 and (E, F) 9 PCW. *AHI1* transcripts are abundant in the mesonephros (Meso) and metanephros (Meta) of the developing kidney as well as the spinal cord (Sc), liver and embryonic gonad. (G, H). A series of sections at different stages of mesonephric and metanephric development. *AHI1* expression is seen in (G) mesonephric excretory unit - a mesonephric tubule, glomerulus and duct at CS14, CS16 and CS22. By CS23 expression is visible in degenerating glomeruli (DG). By 9 PCW expression is detectable in the mesonephric tubule, ducts and paramesonephric duct. *AHI1* expression is seen in (H) early permanent metanephric kidney with intense staining in the metanephric cap and ureteric bud (CS14 and CS16). By CS22 and later developing glomeruli, tubules and collecting ducts are seen to strongly express *AHI1* (CS22, CS23 and 9 PCW). In 9 PCW human fetal sections, *AHI1* transcripts are abundant in the developing nephrons and collecting ducts of renal cortex and there is weak expression in medulla/renal pelvis. CD, collecting duct; DG, degenerating glomerulus; Gl, glomerulus; H, hindgut; LL, lower limb; Mc, metanephric cap; Md, mesonephric duct; Med, renal medulla; Meso, mesonephros; Meta, metanephros; Mt, mesonephric tubule; P, renal pelvis; Pmeso, paramesonephric duct; Sc, spinal cord; St, stomach; UB, ureteric bud; UL, upper limb. Scale bars: A–F = 2 mm; G: CS16 = 500 µm; CS14, CS23 & 9WPC = 250 µm; CS22 = 125 µm; H: CS22 & 9WPC = 500 µm; CS14, CS16 & CS23 = 250 µm.

*AHI1* expression during human nephrogenesis was examined from stages CS14 through to 9 PCW (Figure 2). These studies revealed that *AHI1* transcripts are abundant in both the developing mesonephros and metanephros. Extra renal sites of expression included prominent staining of the embryonic liver and embryonic gonad (Figure 2D, F). Detailed studies through mesonephric development showed discrete *AHI1* expression in the mesonephric excretory unit at CS12 (data not shown) and CS14–CS22 (Figure 2G). By CS23, *AHI1* expression was visible in degenerating glomeruli of the mesonephros and at 9 PCW, expression was detectable in the mesonephric tubule, ducts and paramesonephric duct, structures that will form parts of the future reproductive system (Figure 2G/CS23 and 2G/9PCW respectively). *AHI1* expression was seen in early (permanent) metanephric kidney (Figure 2H) with intense staining in the metanephric cap and ureteric bud at CS14 and CS16. By CS22 and later, developing glomeruli, tubules and collecting ducts strongly expressed *AHI1* (Figure 2H).

More detailed analysis of *AHI1* expression at 9 PCW revealed intense staining in both tubular epithelium and glomerular structures (Figure 2H/9 PCW). Expression was seen in cortical and medullary epithelium, including the epithelium lining of the renal calyces. Glomerular staining was intense in a subset of cells whilst there was an absence of expression in the renal interstitium, indicating low levels of *AHI1* transcript in interstitial cells and vascular endothelium.

Thus, in human tissue *AHI1* is strongly expressed in all developing renal tissues from the mesonephros to the precursors of the adult kidney, with a broad expression throughout the nephron and collecting ducts during renal development.

### *CEP290* expression during human brain and kidney development

The *CEP290* spatial expression during development in human tissue has not previously been reported. Previous characterisation has been limited to hybridisation to RNA northern blot of adult tissues showing expression of a major *CEP290* transcript of approximately 8 kb that is expressed strongly in placenta and weakly in adult brain [3].

**Figure 3:**
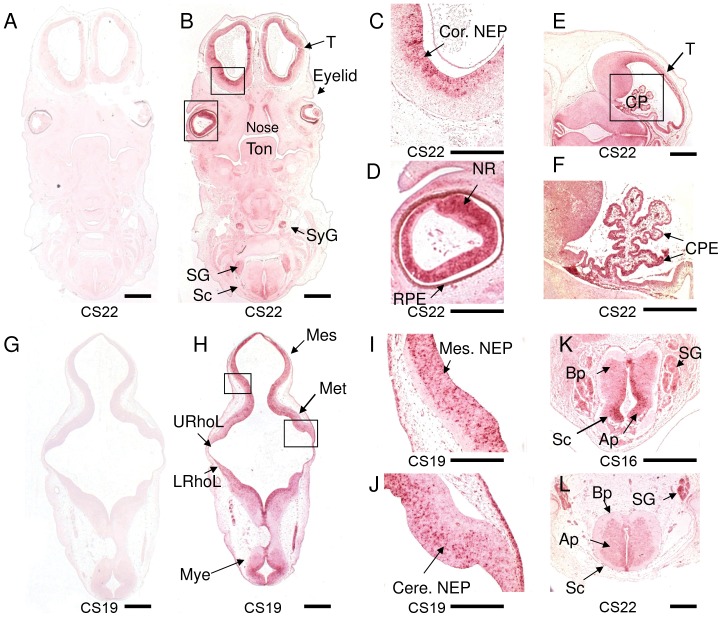
*CEP290* is expressed in the developing cerebellum, spinal cord and eye. (A, G) Negative control hybridisation with *CEP290* sense RNA probe and (B–F) hybridisation with *CEP290* antisense RNA probe to transverse sections at CS22. *CEP290* transcripts are abundant in the developing telencephalon (B, C, E), especially the cortical neuroepithelium (C), and neural retina (D). *CEP290* expression is demonstrated within the epithelium of the choroid plexus (CP) (E, F) at CS22. Prominent expression is seen in metencephalon (including cerebellum), myelencephalon and mesencephalon at CS19 (H, I, J). Strong expression is detected at the rhombic lip in the developing cerebellum (H, J), the mesencephalic (I) and cerebellar neuroepithelium (J). Prominent staining is demonstrated within the alar plate of spinal cord at CS16 (K) and CS22 (L). Ap, alar plate; Bp, basal plate; CP: choroid plexus; CPE, choroid plexus epithelium; Mes, mesencephalon (midbrain); Met, metencephalon; Mye, myelencephalon; NEP, neuroepithelium; Cor.NEP: cortical neuroepithelium; Cere.NEP: cerebellar neuroepithelium; Mes.NEP: mesencephalic (midbrain) neuroepithelium; NR, neural retina; RPE, retinal pigment epithelium, Sc, spinal cord; SG: spinal ganglion; SyG: sympathetic ganglia; T, telencephalon, Ton, tongue; LRhoL: lower rhombic lip; URhoL: upper rhombic lip; Scale bar: A, B, G, H = 2 mm, C, D, F, K = 500 µm; E = 1 mm; L = 500 µm; I, J = 250 µm.

Using specific probes targeted towards nucleotides 6265 to 6924 of *CEP290* mRNA we observed a strong and specific pattern of expression in the brain and spinal cord from CS16 to CS22 (Figure 3). *CEP290* expression was seen in the developing telencephalon (Figure 3B), especially the neuroepithelium, and in the neural retina (Figure 3C, D respectively). Prominent expression was seen in the choroid plexus (Figure 3E, F) and throughout the developing hindbrain (Figure 3H). Expression of *CEP290* is also seen in the rhombic lip of the developing cerebellum (Figure 3H) and the mesencephalic and cerebellar neuroepithelium (Figure 3I, J). In the spinal cord there was particularly prominent staining within the alar plate and some staining of the spinal and sympathetic ganglia (Figure 3K, L). This expression pattern seen in the developing human brain with *CEP290* mirrors the pattern seen for *AHI1* (compare Figure 1 with Figure 3).

**Figure 4:**
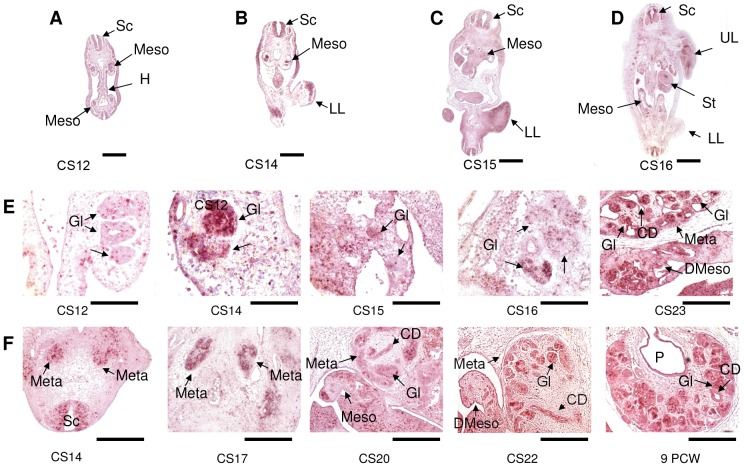
*CEP290* is expressed in the developing kidney. Hybridisation with *CEP290* antisense RNA probe (A–F) to transverse sections. (A) CS12 and (B) CS14; (C) CS15; (D) CS16. *CEP290* transcripts are abundant in the mesonephros (Meso) and metanephros (Meta) of the developing kidney as well as the spinal cord (Sc). (E, F) A series of sections through mesonephric and metanephric development, respectively, from CS14 to 9 PCW. Hybridisations were also carried out with *CEP290* sense probe as a negative control and no signals were detected (data not shown). CD, collecting duct; Gl, glomerulus; H, hindgut; Meso, mesonephros; DMeso, degenerating mesonephros; Meta, metanephros; Sc, spinal cord; St, stomach; UL, upper limb; LL, lower limb. P, renal pelvis. Scale bar: A–D = 1 mm; E: CS12–CS16 = 500 µm; CS23 = 125 µm F: CS 14 = 250 µm; CS17 = 125 µm; CS20 = 250 µm; CS22 and 9 PCW = 125 µm.

*CEP290* expression during human nephrogenesis was examined from CS12 through to 9 PCW (Figure 4). *CEP290* transcripts are abundant in both the developing mesonephros and metanephros (Figure 4E, F). Specific areas of *CEP290* expression are seen in the mesonephric excretory unit at CS12–CS23 (Figure 4E). By 9 PCW, *CEP290* expression was seen in early (permanent) metanephric kidney with intense staining in the developing glomeruli, tubules and collecting ducts (Figure 4F, 4E/9PCW). Thus, like *AHI1, CEP290* is strongly expressed in the mesonephros and metanephros precursors of the adult kidney. Similar to *AHI1*, expression of CEP290 was low/absent in the renal interstitium.

### *AHI1* and *CEP290* are highly evolutionarily conserved and are generally present together in animal genomes

This great similarity of expression in humans prompted us to ask if *AHI1* and *CEP290* might have co-evolved. We have previously shown using a reciprocal BLASTp approach that *AHI1* is highly evolutionarily conserved, and is restricted to the genomes of ciliated organisms [49]. Given the strong overlap in expression between *AHI1* and *CEP290*, we extended this analysis using PSI-BLAST and reciprocal BLASTp to identify additional homologues. We searched the predicted proteomes of 44 eukaryotic organisms chosen to represent a wide evolutionary spread of organisms, both ciliated (33 organisms) and non-ciliated (11 organisms). Both *ahi1* and *cep290* evolved prior to the evolution of multicellularity, are conserved from ciliated protozoa to man, and are restricted to the genomes of organisms that build cilia or flagella (Supplementary Figure S1). Putative orthologues of both *AHI1* and *CEP290* were present in the genomes of organisms that build only sensory cilia (*Daphnia*, *Tribolium*) as well as in the genomes of organisms that build either motile cilia/flagella only, or both motile and sensory cilia, implying that both *ahi1* and *cep290* are required for basal body/cilium function in cells with motile cilia and cells with sensory cilia. Building on our previous observation that *ahi1* was only present in the genomes of organisms that build a canonical nine-triplet centriole [49], both *ahi1* and *cep290* were absent from the genome of *Toxoplasma gondii* and the two nematode genomes in our study, which all build specialised, non-canonical centrioles. Therefore, *ahi1* and *cep290* may both be required for triplet centriole function. In animals, *ahi1* and *cep290* were both found in all organisms except in Dipthera (*Anopheles* and *Drosophila*) and *Gallus*, indicating a possible common function in metazoa that is consistent with the expression data. However, while there was a general trend for *ahi1* and *cep290* to both be present in a given genome, this was not invariable. We found only a predicted *AHI1* orthologue in 5 genomes (*Aureococcus anophagefferens, Naegleria gruberi,, Trichomonas vaginalis, Trypanosoma brucei*) and only a predicted *CEP290* orthologue in a further 5 genomes (*Anopheles gambiae, Drosophila melanogaster, Micromonas pulsilla, Monosiga brevicollis, Gallus gallus*) suggesting that, while *ahi1* and *cep290* might function together in animals, they are not co-dependent in all organisms.

### Cep290 does not require Ahi1 for localisation

**Figure 5:**
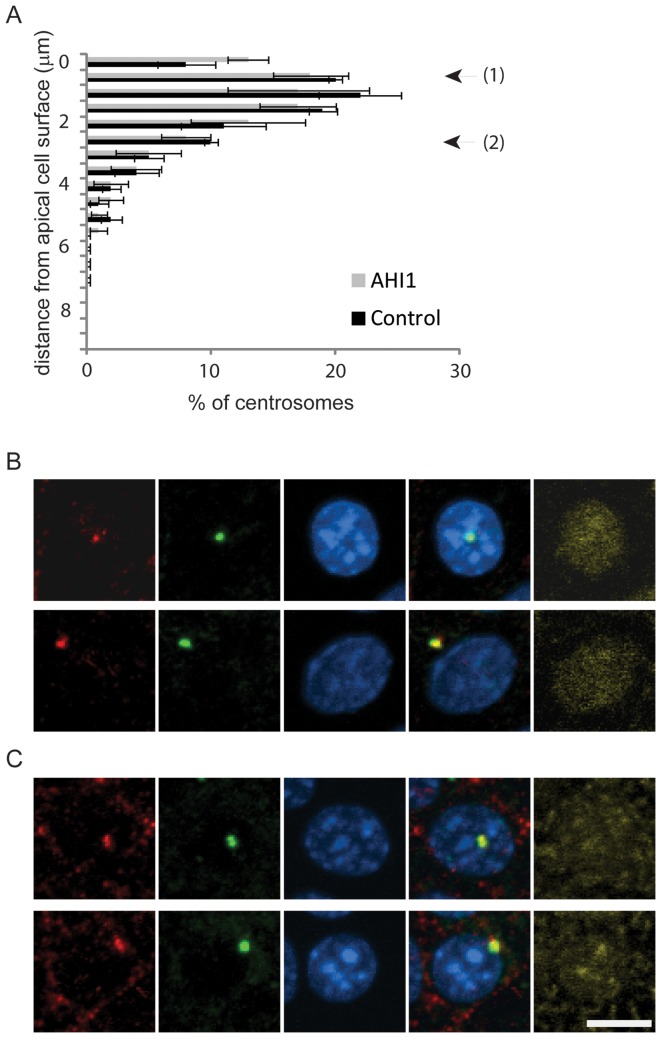
Cep290 does not require Ahi1 for localisation to centrosomes. Immunofluorescence of IMCD3 monolayers co-transfected with siGLO and either negative-control siRNA (A and B), or siRNA against *Ahi1* (A and C). (A) Graph to show centrosome position (gamma tubulin immunofluorescence, graph expressed as mean +/− s.e.m.) from apical to basal in control and *Ahi1*-silenced cells. Apical refers to the upper 1.5–2 µm of the cell (the distance between the nuclear envelope and the plasma membrane in these cells; this is unchanged following silencing of *Ahi1*, data not shown). Arrows denote the apical and mid-cell positions used in B and C. (B and C) Confocal maximum intensity projections of control (B) and *Ahi1*-silenced (C) cells showing Cep290 (red), gamma tubulin (green) and DNA (blue). Transfected cells were identified using siGLO (yellow). In each case, the top panel represents an apically-oriented centrosome (position 1 on the graph, A) and the bottom panel represents a centrosome from the mid-cell region (position 2 on the graph). Scale bar: 5 µm.

Cep290 is a 290 kDa protein that is localised at the centrosome [3], [36], [42], [51]. To examine whether Ahi1 is necessary for correct recruitment of Cep290 to centrosomes [52], [53], we ablated *Ahi1* expression using siRNA in IMCD3 renal epithelial cells [49] and visualised Cep290 and centrosomes using anti-Cep290 and anti-gamma tubulin immunofluorescence. We examined the appearance of the Cep290 signal at centrosomes throughout the cell. There was no difference in the distribution of centrosomes between *Ahi1*-silenced cells and controls (Figure 5A), indicating that the centrosome is still able to re-orient to the apical cell surface in the absence of Ahi1. We found no obvious differences in the immunofluorescence pattern between control and *Ahi1* siRNA-treated cells (Figure 5B and C), regardless of the centrosome position within the cell, indicating that Ahi1 and Cep290 are not interdependent for localisation to centrosomes.

## Discussion

We have demonstrated early embryonic expression of *AHI1* and *CEP290* in human embryonic tissues, with a remarkable similarity in expression patterns. Both *AHI1* and CEP290 are widely expressed in multiple tissues, including developing brain, eye, spinal cord and renal tissues. Previous expression studies in murine tissues showed *Ahi1* expression in neurons of the developing hindbrain/midbrain/forebrain, pituitary, testis and kidney [50]. *Ahi1* expression in murine brain was reported during early embryonic life (day 10.5) and persisted into adult life. Interestingly, in Doering's studies, mouse Ahi1 protein expression was not seen in the developing or mature cerebellum although AHI1 was present in the human cerebellum, suggesting a species difference [50]. Indeed, these studies commented that the highest AHI1 expression in adult brain tissue was in the cerebellar vermis and cerebellar peduncles [50]. A functional role for *AHI1* in brain development is suggested by the numerous structural defects associated with Joubert syndrome [13], [14].

We were able to demonstrate prominent *AHI1* expression in the developing retina. It is now well established that the light sensitive photoreceptor cells of the retina are specialised sensory cilia. Inherited forms of retinal degeneration, where these cells are affected are now a hallmark of diseases termed ciliopathies [54], [55]. Numerous gene defects, encoding proteins expressed in cilia, may be associated with retinal degeneration. *NPHP5* mutations are a prominent cause of retinal degeneration [56]. *Ahi1^−/−^* mutant mice fail to develop photoreceptor outer segments, leading to retinal degeneration [31], [57].

Ahi1 was seen to be expressed at the transition zone of photoreceptor cells of wild-type mice, colocalising with the transition zone marker Rpgrip1 [57]. The *Ahi1−/−* murine model mimics closely the defect seen in JSRD patients with *AHI1* mutations [7], [9], [30]. Our data of human *AHI1* expression in retinal development confirms this requirement of AHI1 for normal photoreceptor development.

Consistent with the renal phenotype seen in some JSRD patients [29], we demonstrate for the first time *AHI1* and *CEP290* expression in the human embryonic kidney. Indeed, murine data showing detailed kidney expression of these genes is also lacking. The pattern of expression during development of the mesonephros and metanephros points to a role in tubulogenesis, epithelial cell polarity and maintenance of orientated cell division, as has been described for other ciliary proteins [58].

In human adult kidney, we have previously demonstrated that Ahi1 protein expression is limited to the distal tubule and collecting duct nephron segments [32], whilst murine kidney tissue from a 5 month week old animal revealed *Ahi1* expression at the corticomedullary junction [59]. A postnatal increase in Ahi1 protein expression has also been described [59]. This suggests that renal Ahi1 expression must be regulated throughout development and into adult life, and that a role for maintenance of distal tubular function continues into adult life.

There have been no previous studies documenting the renal expression of *CEP290* in human kidney. Previous studies have documented expression of murine *Cep290,* with RNA blot analysis revealing expression at embryonic day 7 to E17 in whole embryos, and kidney and brain expression at P0. [36]. *In situ* hybridisation studies in murine cerebellum have demonstrated expression in the external (proliferative) and internal granule layer in the midline at E18 and the latter expression persists into adulthood [36]. At the earlier human stages studied here, *CEP290* expression is detected in cerebellar neuroepithelium, also a proliferative layer, as well as mesencephalic and telencephalic neuroepithelium.

The Cep290 protein has also been localized within photoreceptor cells, to the connecting cilium adjoining the outer segment to the photoreceptor cell body [3], [60].

*CEP290* mutations are the commonest monogenic form of Leber's congenital amaurosis (LCA) [61]. A murine model of *Cep290* deficiency reveals early post-natal photoreceptor degeneration [60] whilst human studies in patients with *CEP290* mutations suggest that whilst there is initial development of rod photoreceptors during early development, the distal segments of photoreceptors are structurally abnormal after maturation, causing early and severe loss of visual function typically within the first decade of life [61]. The retinal expression of *CEP290* we have shown during early development in humans is consistent with a role for *CEP290* in photoreceptor cell development.

The expression of both *AHI1* and *CEP290* in the choroid plexus is a novel and interesting finding. The choroid plexus produces cerebrospinal fluid and is circulated through the ventricles of the brain and the subarachnoid space. The ependymal cells lining the ventricular space are known to possess motile cilia, and that defects in these motile cilia can contribute to an obstruction of CSF flow leading to hydrocephalus [62], [63], [64], [65]. In contrast, the murine ciliopathy model *Tg737^orpk^* demonstrated hydrocephalus before formation of motile cilia in the ependymal cells [66], implicating defects in choroid plexus function (such as increased CSF production) as a primary event in these animals. Primary cilia are intimately related to the regulation of CSF production. Indeed clusters of primary cilia on choroid plexus epithelia cells have been shown to act as chemosensors to regulate CSF production [67]. Upon *ahi1* knockdown in zebrafish, we have previously shown hydrocephalus, alongside other defects associated with a ciliopathy [49]. A similar appearance, with prominent hydrocephalus was seen with cep290 (alias *nphp6*) knockdown in zebrafish [68]. To date, murine models of *Ahi1* and *Cep290* knockdown have not demonstrated gross hydrocephalus. The brain defect that these mice have revealed includes cerebellar hypoplasia with a vermis/midline fusion defect early in development [69].

In humans with JSRD, hydrocephalus is a rarer finding, however recently mutations in the *CC2D2A* gene (whose protein product interacts with CEP290 [18]) have been associated with ventriculomegaly and hydrocephalus [70]. Our experiments suggest that a functional role for *AHI1* and *CEP290* is likely within the developing brain, including the choroid plexus.

A proteomics and interaction mapping project has recently been published which details a number of important interactions for both Ahi1 and Cep290 [71]. Mks1, Mks6, and Tectonic1 bind to Ahi1 protein, which also co-purified with inversin, whilst Cep290 bound strongly with nephrocystin-5. Depletion of nephrocystin-5 had no effect on Cep290 localization, whilst nephrocystin-5 failed to localize to the centrosome in the absence of Cep290 [71]. The studies defined 3 protein “connecting modules” where nephrocystin proteins −1, −4 and −8 function together at the apical surface of the cell, nephrocystin-5 and -6 function at the centrosome and the MKS group of proteins (Mks1, Mks6 and Tctn2) function in a hedgehog signalling pathway [71]. Ahi1 was seen as a linking protein between MKS and centrosomal modules. In an attempt to determine whether Ahi1 is necessary for correct recruitment of Cep290 to the area around centrosomes, we found no loss of localisation, which is consistent with Sang's recently published localisation data set [71].

In conclusion, we have demonstrated in human development an expression pattern for *AHI1* and *CEP290* which includes neuronal, retinal and renal structures. *AHI1* and *CEP290* are highly conserved throughout evolution, but can be expressed and function independently.

## Supporting Information

Supplementary Figure S1 **Distribution of ahi1 and cep290 proteins and cilium and centriole architecture across eukaryotes.** Putative ahi1 and cep290 homologues are found in organisms that build both motile and sensory cilia. Both are present in most animals; however ahi1 and cep290 can also occur individually in organisms. Ultrastructural information was not available for all organisms included in this study. A ? denotes an unknown architecture or one where conflicting data have been reported. Architectures are in accordance with those described in Simms *et al*. [49].
(TIF)
