# Supplementary figures and images for: Investigating Embryonic Expression Patterns and Evolution of AHI1 and CEP290 Genes, Implicated in Joubert Syndrome
Source: PLoS One. 2012 Sep 24;7(9):e44975. doi: 10.1371/journal.pone.0044975 (PMC3454386; doi:10.1371/journal.pone.0044975)

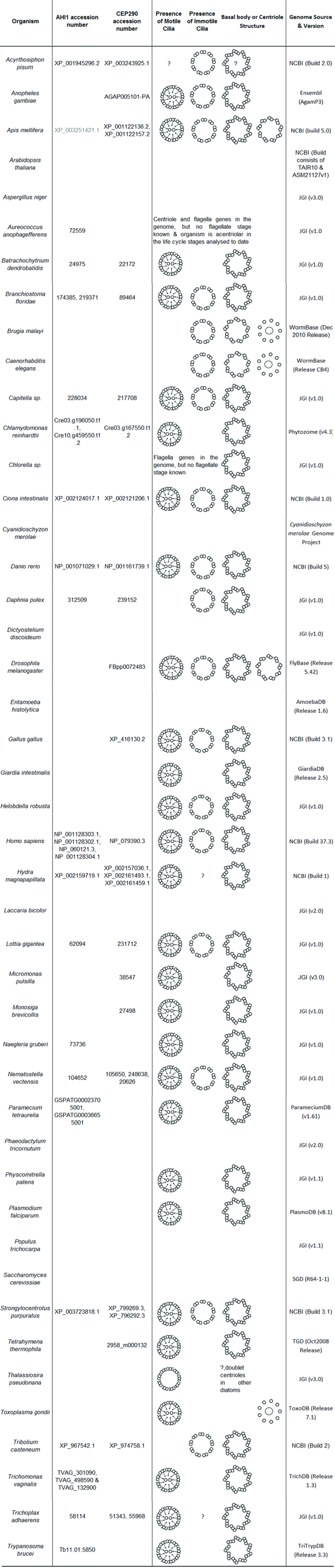

Supplement: Supplementary Figure S1 — Distribution of ahi1 and cep290 proteins and cilium and centriole architecture across eukaryotes. Putative ahi1 and cep290 homologues are found in organisms that build both motile and sensory cilia. Both are present in most animals; however ahi1 and cep290 can also occur individually in organisms. Ultrastructural information was not available for all organisms included in this study. A ? denotes an unknown architecture or one where conflicting data have been reported. Architectures are in accordance with those described in Simms et al. [49]. (TIF) [file pone.0044975.s001.tif]
